# Metapopulation dynamics in a changing climate: Increasing spatial synchrony in weather conditions drives metapopulation synchrony of a butterfly inhabiting a fragmented landscape

**DOI:** 10.1111/gcb.14280

**Published:** 2018-05-16

**Authors:** Aapo Kahilainen, Saskya van Nouhuys, Torsti Schulz, Marjo Saastamoinen

**Affiliations:** ^1^ Metapopulation Research Centre, Organismal and Evolutionary Biology Research Programme Faculty of Biological and Environmental Science University of Helsinki Helsinki Finland; ^2^ Department of Entomology Cornell University Ithaca New York

**Keywords:** climate change, dispersal, Lepidoptera, life history, *Melitaea cinxia*, metapopulation dynamics, population synchrony, precipitation, temperature, trophic interactions

## Abstract

Habitat fragmentation and climate change are both prominent manifestations of global change, but there is little knowledge on the specific mechanisms of how climate change may modify the effects of habitat fragmentation, for example, by altering dynamics of spatially structured populations. The long‐term viability of metapopulations is dependent on independent dynamics of local populations, because it mitigates fluctuations in the size of the metapopulation as a whole. Metapopulation viability will be compromised if climate change increases spatial synchrony in weather conditions associated with population growth rates. We studied a recently reported increase in metapopulation synchrony of the Glanville fritillary butterfly (*Melitaea cinxia*) in the Finnish archipelago, to see if it could be explained by an increase in synchrony of weather conditions. For this, we used 23 years of butterfly survey data together with monthly weather records for the same period. We first examined the associations between population growth rates within different regions of the metapopulation and weather conditions during different life‐history stages of the butterfly. We then examined the association between the trends in the synchrony of the weather conditions and the synchrony of the butterfly metapopulation dynamics. We found that precipitation from spring to late summer are associated with the *M. cinxia* per capita growth rate, with early summer conditions being most important. We further found that the increase in metapopulation synchrony is paralleled by an increase in the synchrony of weather conditions. Alternative explanations for spatial synchrony, such as increased dispersal or trophic interactions with a specialist parasitoid, did not show paralleled trends and are not supported. The climate driven increase in *M. cinxia* metapopulation synchrony suggests that climate change can increase extinction risk of spatially structured populations living in fragmented landscapes by altering their dynamics.

## 1 INTRODUCTION

The two most prominent manifestations of human‐induced global change are habitat loss with habitat fragmentation and climate change. Although the former continues to be the main causative agent in driving species extinctions (Millennium Ecosystem Assesment, 2005; Newbold et al., 2015; Pimm et al., 2014; Tittensor et al., 2014), the effects of climate change are expected to increase to matching levels during the coming decades (Leadley et al., 2010). With the rapid advance of both, one of the most central questions in the contemporary research on biodiversity, conservation and ecology is how these two facets of global change jointly influence natural populations (Eigenbrod, Gonzalez, Dash, & Steyl, 2015; Holyoak & Heath, 2016; Mantyka‐Pringle, Martin, & Rhodes, 2012; McGill, Dornelas, Gotelli, & Magurran, 2015; Oliver & Morecroft, 2014). However, as detailed long‐term data on the dynamics of spatially structured metapopulations in fragmented landscapes are available for only a few systems, the exact mechanisms by which climate change modifies the effects of habitat fragmentation are not well understood (Holyoak & Heath, 2016; Oliver & Morecroft, 2014). Moreover, studies on the joint effects of habitat fragmentation and climate change primarily focus on changes in the mean climatic conditions. Changes in variability of weather conditions can also influence populations in fragmented landscapes, and variability is likely to change as climate change advances. Information on how changes in climatic variability can influence populations inhabiting fragmented landscapes is of utmost importance (Alexander & Perkins, 2013; Easterling, 2000; Huntingford, Jones, Livina, Lenton, & Cox, 2013; IPCC, 2013; Lawson, Vindenes, Bailey, & van de Pol, 2015).

Metapopulations inhabiting fragmented landscapes are likely very vulnerable to changes in variability of weather conditions. The long‐term stability of a metapopulation relies on independent population dynamics in different parts of the landscape, such that decreases in abundance at one region are balanced out by increases in another, and colonization of unoccupied habitat continuously makes up for local extinctions (Hanski, 1999; Hanski & Woiwod, 1993; Hastings & Harrison, 1994; Heino, Kaitala, Ranta, & Lindström, 1997). The independence of population dynamics over the landscape can be challenged by climate change if climatic conditions are related to population growth rates. Below, we will briefly outline the mechanisms that can disrupt independence and introduce synchrony in population dynamics and how they might be influenced by climate change.

Independence of population dynamics in different parts of a metapopulation can be disrupted by three primary mechanisms: (1) increasing spatial extent of synchrony in environmental conditions influencing population growth rate (i.e., Moran effect; Moran, 1953), (2) increased dispersal of individuals between local populations, and (3) a change in the spatial extent of trophic interactions (e.g., a predator with a geographic range different from that of the prey) (Liebhold, Koenig, & Bjørnstad, 2004). Climate change can drive metapopulation synchrony via any of the above mentioned mechanisms. First, climate change can create a Moran effect via a decrease in spatial environmental variability (Allstadt, Liebhold, Johnson, Davis, & Haynes, 2015; Koenig & Liebhold, 2016; Liebhold et al., 2004; Post & Forchhammer, 2004; Ranta, Kaitala, & Lindstrom, 1999). Second, as temperature and wind conditions influence the dispersal propensity of many taxa (Cormont et al., 2011; Kuussaari, Rytteri, Heikkinen, Heliölä, & von Bagh, 2016), increasing temperature has the potential to drive increasing dispersal resulting in phase locking (Fox, Vasseur, Hausch, & Roberts, 2011; Gyllenberg, Söderbacka, & Ericsson, 1993). Third, climate change can alter the spatial extent of trophic interactions influencing the dynamics of the system, for example, by enabling colonization of new species preying on the focal population or changing the dynamics of existing predator populations. Although we are unaware of studies explicitly documenting the latter case, there are several examples of distribution changes in top predators and changes in the interactions within food chains due to climate change (Gilman, Urban, Tewksbury, Gilchrist, & Holt, 2010; Harley, 2011; Hazen et al., 2012; Romo & Tylianakis, 2013), all of which can disrupt metapopulation dynamics by changing the density‐dependence structure of the prey populations in a given system. Lastly, all the above‐described mechanisms may respond to climate change simultaneously or interact with each other. If all the mechanisms are not considered, the driver of population dynamics synchrony can be misidentified. Therefore, to understand the role of climate change, it is crucial that all of the potential mechanisms are considered.

The Glanville fritillary butterfly (*Melitaea cinxia*) metapopulation in the Finnish archipelago and its associated parasitoids have been extensively studied for over two decades with the aim of understanding habitat fragmentation and metapopulation dynamics (Hanski & Ovaskainen, 2000; Nieminen, Siljander, & Hanski, 2004; Ojanen, Nieminen, Meyke, Pöyry, & Hanski, 2013). A temporal increase in the coherence of the *M. cinxia* metapopulation dynamics was reported by Hanski and Meyke (2005) and Tack, Mononen, and Hanski (2015). Increasing frequency of late summer drought events was suggested as the potential driver of the change, but no change in climatic variability was detected (Tack et al., 2015). Since climate change is expected to have greater influence on spring than on summer conditions in the northern hemisphere, the previous study may have missed important aspects of climate change by focusing solely on late summer precipitation conditions (Bonsal, Zhang, Vincent, & Hogg, 2001; Huntingford et al., 2013; Robeson, 2004). Finally, other potential explanations, such as increasing dispersal, or changes in predation, have not been addressed.

Here, using data on the metapopulation dynamics of *M. cinxia* from 1993 to 2015 together with monthly weather data, we examine the association of synchrony of the metapopulation dynamics of *M. cinxia* with climate. More specifically, we focus on answering the following questions: (i) How has the spatial extent of synchrony of population growth rate changed across time? (ii) How are different weather conditions over the entire life cycle associated with *M. cinxia* population growth rate across different regions of the metapopulation? (iii) Has spatial synchrony of the influential weather conditions changed with time, and is the synchrony of weather associated with metapopulation synchrony? (iv) Can changes in the synchrony of the *M. cinxia* metapopulation be attributed to changes in dispersal propensity or trophic interactions? Our results shed light on the potential mechanisms by which climate change can alter metapopulation dynamics in a fragmented landscape, and thus contributes to the understanding of the potential interaction between habitat fragmentation and climate change.

## 2 MATERIALS AND METHODS

### 2.1 The Finnish Glanville fritillary butterfly metapopulation

The Glanville fritillary butterfly, *M. cinxia*, inhabits a large network of ca. 4400 dry meadows containing at least one of its host plants, ribwort plantain (*Plantago lanceolata*) or spiked speedwell (*Veronica spicata*: Plantaginaceae) in the Åland islands on the southwestern coast of Finland (Nieminen et al., 2004). The Finnish *M. cinxia* metapopulation is univoltine. Its development can be divided to egg, prediapause larval (instars from 1st to 4th/5th), postdiapause larval (instars from 4th/5th to 7th), chrysalis, and adult stages (Kuussaari, van Nouhuys, Hellemann, & Singer, 2004; Murphy, Wahlberg, Hanski, & Ehrlich, 2004; Wahlberg, 2000). Based on our observations, the first adults normally emerge in late May or early June. The flight season lasts approximately 30 days (mean 30 days, median 32 days; Table S1), ending by early July. The first prediapause larval nests start appearing in mid‐July, first overwintering silk nests (4th or 5th instar) can be found in mid‐August, and most nests have entered diapause by the beginning of September. The larvae diapause until late March, after which they go through from two to three additional larval stages before pupation in mid‐May (Kuussaari et al., 2004; Saastamoinen, Ikonen, Wong, Lehtonen, & Hanski, 2013; Wahlberg, 2000).

Since 1993, the suitable meadows in Åland have been censused for *M. cinxia* occupancy and population size by counting the number of overwintering larval nests every autumn, followed up by a check of overwintering mortality of the nests the following spring (Nieminen et al., 2004; Ojanen et al., 2013). The initial number of surveyed habitat patches was ca. 1200. Then, between 1998 and 1999 an extensive remapping of potential habitats was conducted, after which the number of surveyed patches increased threefold. Currently, ca. 4400 habitat patches are surveyed (Hanski et al., 2017; Ojanen et al., 2013). Estimates of the detection probability of each overwintering nest varies between 0.5 and 0.6, but the probability of incorrectly inferring a habitat patch as unoccupied is only 0.1 (Ojanen et al., 2013). Typically, the undetected populations are small, consisting of one or very few larval nests, so their contribution to the metapopulation dynamics is negligible. For the few cases in which more nests were observed in the spring than in the previous autumn, the autumn nest count has been corrected to match that of the spring nest count. Other than changes in the number of habitat patches surveyed, the changes to the systematic survey protocol and sampling effort have been minor throughout the years, which makes observations across years comparable (Ojanen et al., 2013).

Due to aggregation of habitat patches in the landscape, the Åland islands can be subdivided into semi‐independent habitat patch networks (SINs), with habitat connectivity that is high enough to allow for frequent exchange of dispersing individuals between patches within the same SIN (Hanski, Moilanen, Pakkala, & Kuussaari, 1996). Using hierarchical clustering implemented in the software SPOMSIM (Moilanen, 2004), a recent study clustered the entire metapopulation to 125 SINs that differ in patch number, size and connectivity (Hanski et al., 2017). Of these, 33 SINs can be considered viable according to spatially explicit metapopulation theory (i.e., metapopulation capacities above a species specific extinction threshold; Hanski et al., 2017). Each of the viable SINs contain on average 82 habitat patches (median = 69, *SD* = 39) with an average area of a patch of 2260 m^2^ (median = 687 m^2^, *SD* = 5467 m^2^). The viable SINs are distributed throughout the Åland islands (Hanski et al., 2017).

In the present study, we chose to focus on the dynamics of SINs rather than on individual habitat patches. We do so because local populations in individual habitat patches frequently go extinct, so time series of local population growth rate dynamics would be very heterogeneous. Furthermore, the spatial scale of our weather data better matches the spatial scale of the SINs rather than the individual habitat patches. We exclude the nonviable SINs from the analyses because many of them are unoccupied for all or most of the 23‐year study period.

### 2.2 Natural enemies of *M. cinxia*

*Melitaea cinxia* has been observed to be host to a generalist pupal parasitoid species *Pteromalus apum*, and prey to lady beetles (Coccinellidae), lacewings (Chrysopidae), pentatomid bugs (Pentatomidae), red ants (*Myrmica rubra*), spiders, and dragonflies (Odonata) (van Nouhuys & Hanski, 2004; van Nouhuys & Kraft, 2012). The rate of predation by these generalists has not been systematically recorded, however, we do not expect them to have greatly impacted the synchrony of population dynamics of the host because we have observed no evidence of large changes in predator community over time, and mobile individuals of these taxa would not be likely to track *M. cinxia* density in the landscape. Furthermore, *M. cinxia* are chemically defended by sequestered plant defensive chemicals (Reudler & van Nouhuys, 2018; Suomi, Sirén, Jussila, Wiedmer, & Riekkola, 2003) and are thus not likely to be prey to many invertebrate and vertebrate predators (Kuussaari et al., 2004).

While we do not expect a large role for generalist predators, *M. cinxia* larvae are frequently parasitized by two specialist parasitoid wasp species, *Cotesia melitaearum* (Braconidae: Microgastrinae) and *Hyposoter horticola* (Ichneumonidae: Campoplaginae) (van Nouhuys & Hanski, 2004, 2005). Of the two species, only *C. melitaearum* has the potential to influence synchrony of the *M. cinxia* metapopulation dynamics. This is because the highly mobile *H. horticola* invariably parasitizes one third of the caterpillars in almost every nest every year across the metapopulation (Montovan, Couchoux, Jones, Reeve, & van Nouhuys, 2015; van Nouhuys & Ehrnsten, 2004; van Nouhuys & Hanski, 2002). The sedentary *C. melitaearum*, on the other hand, is restricted to the northwestern side of the archipelago in most years and inhabits only well‐connected *M. cinxia* SINs (van Nouhuys & Hanski, 2002). Where present, *C. melitaearum* can be locally abundant, potentially driving local populations of *M. cinxia* to extinction (Lei & Hanski, 1997). Furthermore, rate of parasitism and subsequent parasitoid population size is related to spring temperature (van Nouhuys & Lei, 2004). The *M. cinxia* populations are surveyed for *C. melitaearum* every spring when the mortality of the overwintering nests is surveyed (Ojanen et al., 2013). At this time, the overwintering generation of the *C. melitaearum* larvae leave the host and spin white silken cocoons that are visible in the *M. cinxia* nests (van Nouhuys & Lei, 2004). Although otherwise spanning the entire study period, the data for the occurrence of *C. melitaearum* in 2010 was unfortunately lost due to a hard drive break down.

### 2.3 Weather data

The weather data used in the analyses is a part of ClimGrid, which is a gridded climatology dataset with a cell size of 10 km * 10 km (Aalto, Pirinen, & Jylhä, 2016), provided by the Finnish Meteorological Institute. We obtained monthly average temperature and precipitation sum estimates for months that approximately match the different life‐history stages of *M. cinxia* (see above), for the area covering the Åland islands (from 59.9° to ca. 60.5° Lat. and from ca. 19.5° to ca. 20.9° Lon.). Weather conditions from September to the following February were averaged to reflect average conditions during diapause and, for this period, we also extracted average snow cover depth and incorporated it into analyses of population growth rate (see below). These analyses can be found in the supplementary material, but for simplicity, we will restrict our focus to precipitation and temperature in the main text. For the rest of the weather conditions, we considered each month separately: March, April, and May were considered to reflect postdiapause larval conditions, June conditions to reflect the adult stage conditions (Table S1), and July and August conditions to reflect prediapause larval conditions (Kuussaari et al., 2004; Murphy et al., 2004). It is more difficult to associate egg and pupal stages with any particular month as they last a shorter period of time and overlap with the timing of larval and adult stages. The egg stage mostly coincides with the adult stage in June, but can partly coincide with prediapause larval stages in early July, and the pupal stage occurs mostly in May coinciding with late instars of postdiapause larval development (Murphy et al., 2004).

As nonstationarity due to temporal trends in time series data may bias analyses (Bjørnstad, Ims, & Lambin, 1999; Liebhold et al., 2004; Legendre & Legendre, 2012; but see Chevalier, Laffaille, Ferdy, & Grenouillet, 2015), we tested for temporal trends in each of the weather variables and detrended the variables whenever trends were detected. We estimated the number of smooth temporal basis functions using the R package SpatioTemporal (Lindström, Szpiro, Sampson, Bergen, & Oron, 2013), by fitting up to five smooth orthogonal basis functions in addition to an intercept model. The appropriate number of functions was selected based on BIC values obtained via cross‐validation, and the selected functions were then used as covariates in linear regressions to withdraw residuals that were then used as detrended weather variables.

### 2.4 Associations between growth rate and weather conditions

We calculated the log‐transformed population growth rates for each viable SIN for each year as *r*_*i,t*_ = log [(*N*_*i,t*_ + 1)/(*N*_*i,t*‐1_ *+* 1)], where *N*_*i,t*_ is the number of overwintering larval nests in SIN *i* at time *t*. For simplicity, we refer to the log‐transformed population growth rate simply as population growth rate throughout the manuscript.

To examine during which life‐history stages are weather conditions most influential for the population growth rates, we built a set of Bayesian linear mixed models, each corresponding to different biological hypotheses for the influence of weather conditions on population growth rate (Table 1). Each model, excluding the null model, included a set of detrended weather covariates corresponding to different life‐history stages or a combination of them. Since the habitat patches in a single SIN can fall into several different weather data grid cells, weather covariates for each SIN were calculated as the weighted average of the weather conditions of the individual habitat patches. The weights for each patch were obtained from the spatially explicit metapopulation model, by estimating the contribution of the patch to the metapopulation capacity of the SIN based on the spatial location, size, and quality of the habitat patch (for details, see Hanski et al., 2017). Also, as the different weather variables vary at different scales, we standardized them to a mean of zero and unit variance for the analyses. SIN identity was added as a random intercept and, to account for density dependence in the growth rate (Nieminen et al., 2004), we included a first‐order autocorrelation term for population growth rate in all of the models. In addition to the models reported in Table 1, we analyzed a version of model 1 that also included average snow cover depth (Table S2).

**Table 1:** Models for hypotheses regarding the relationship between SIN growth rate and weather. The table includes the covariates, the LOOIC value, and the standard error of the LOOIC for each model

No.	Hypothesis	Covariates	LOOIC	SE
1	Full	T_D_+T_Mar_+T_Apr_+T_May_+T_Jun_+T_Jul_+T_Aug_+P_D_+P_Mar_+P_Apr_+P_May_+P_Jun_+P_Jul_+P_Aug_	1977.26	44.00
2	Diap., postdiap. & adult	T_D_+T_Mar_+T_Apr_+T_May_+T_Jun_+P_D_+P_Mar_+P_Apr_+P_May_+P_Jun_	2002.33	43.27
3	Diap., adult & prediap.	T_D_+T_Jun_+T_Jul_+T_Aug_+P_D_+P_Jun_+P_Jul_+P_Aug_	2016.91	44.04
4	Postdiap., adult, prediap.	T_Mar_+T_Apr_+T_May_+T_Jun_+T_Jul_+T_Aug_+P_Mar_+P_Apr_+P_May_+P_Jun_+P_Jul_+P_Aug_	1976.47	43.17
5	Diap. & postdiap.	T_D_+T_Mar_+T_Apr_+T_May_+P_D_+P_Mar_+P_Apr_+P_May_	2019.64	42.64
6	Diap. & adult	T_D_+T_Jun_+P_D_+P_Jun_	2106.96	43.55
7	Diap. & prediap.	T_D_+ T_Jul_+T_Aug_+P_D_+P_Jul_+P_Aug_	2025.19	44.43
8	Postdiap. & adult	T_Mar_+T_Apr_+T_May_+T_Jun_+P_Mar_+P_Apr_+P_May_+P_Jun_	2002.93	43.81
9*	Post‐ & prediap.	T_Mar_+T_Apr_+T_May_+T_Jul_+T_Aug_+P_Mar_+P_Apr_+P_May_+P_Jul_+P_Aug_	1988.70	42.69
10	Adult & prediap.	T_Jun_+T_Jul_+T_Aug_+P_Jun_+P_Jul_+P_Aug_	2027.23	42.18
11	Diap.	T_D_+P_D_	2110.65	43.69
12	Postdiap.	T_Mar_+T_Apr_+T_May_+P_Mar_+P_Apr_+P_May_	2030.82	42.87
13	Adult	T_Jun_+P_Jun_	2105.49	42.57
14	Prediap.	T_Jul_+T_Aug_+P_Jul_+P_Aug_	2035.86	42.73
15	Temperature	T_D_+T_Mar_+T_Apr_+T_May_+T_Jun_+T_Jul_+T_Aug_	2026.02	43.85
16	Precipitation	P_D_+P_Mar_+P_Apr_+P_May_+P_Jun_+P_Jul_+P_Aug_	1999.48	44.69
17	Null	Random intercept and autocorrelation only	2112.83	42.44

*P*_D_: average diapause period precipitation; *P*_Mon:_ monthly precipitation; *T*_D_: average diapause period temperature; *T*_Mon_: monthly average temperature.

We then conducted model selection based on the “leave‐one‐out” information criterion (LOOIC; Vehtari, Gelman, & Gabry, 2016) to choose the most informative model(s) for further inspection and to be used in downstream analyses of the synchrony of weather conditions.

### 2.5 Temporal trends in the metapopulation and weather synchrony

For estimating how the spatial extent of synchrony in population growth rates has changed with time across the entire metapopulation, we divided the data into seven 5‐year time periods, each overlapping the previous one by 2 years. The only exception is the last time window from 2012 to 2015, which covers only 4 years. Shifting the frame of the time windows such that the first one contains 4 years (i.e., 1994–1997) instead of the last one does not change the results (results not shown). Within each of these time windows, we counted pairwise cross‐correlations in population growth rate between all pairs of SINs, transformed them to Fisher's *z* to account for the truncated distribution, and divided them into distance bins with 10 km increments to match the resolution of the weather data. We then withdrew average *z*‐transformed cross‐correlations and estimated the standard error of the average pairwise correlation from 1000 bootstrapped datasets. In each dataset, each SIN was represented only once to avoid pseudoreplication (Koenig & Knops, 1998).

For the detrended weather variables, we calculated synchronies and their confidence intervals in different distance classes in each time window, similar to that done for the SIN growth rate (see above). When conducting the Fisher's *z* transformation to the weather variables, cross‐correlations with a value of one were removed from the data. Such cases appeared only in the temperature variables and represent only 0.2% of all pairwise cross‐correlations. We then combined the individual synchrony estimates to obtain an estimate of overall synchrony in weather conditions that are central for *M. cinxia* SIN growth rates. For this, we calculated the weighted median across the synchronies in different weather variables, using the absolute values of the coefficients obtained from the selected linear model of the relationship between SIN growth rate and weather variables (see above). We chose to use median to avoid overestimation of the correlation due to some extreme values. Combining *z*‐transformed correlation values describing synchrony in different weather variables comes with the difficulty that they differ in their overall levels and ranges. Hence, variables that have wider ranges of variability would dominate after combining, which would complicate the biological interpretation of the combined correlation. Therefore, we standardized the *z*‐transformed pairwise correlations to a zero mean and unit variance for each weather variable prior combining them.

To verify the temporal and distance trends in growth rate and weather synchrony, we ran Bayesian linear models with time window, distance class and their interaction as covariates (see details in “2.8 Implementation of statistical analyses”). As the synchrony estimates in each distance class in each time window are averages or medians of pairwise Fisher's *z*‐transformed Pearson correlations between SINs or raster cells, we incorporated the standard error in the response to the linear models (for estimating standard error, see above). To account for temporal autocorrelation between consecutive time windows in the above‐described analyses, the models were run with a first‐order autocorrelation term.

In order to study the association between population growth rate and weather synchronies, we derived estimated residual synchronies and their standard errors from the above‐described models and used them in a Bayesian linear model accounting for error in both the response (residual population growth rate synchrony) and the predictor (residual weather synchrony). We focused on residuals instead of raw variables in order to obtain a robust estimate of the association avoiding including any spatial and/or temporal trends in the estimates of the association between the two synchronies. As the association between the two synchronies can differ in different distance classes, we included distance class and its interaction with weather synchrony in the analysis.

### 2.6 Temporal trends in connectivity and colonization dynamics

As we do not have annual mark–recapture data spanning the entire metapopulation, our data do not contain direct estimates of dispersal between habitat patches. However, we do have annual data on habitat patch occupancies, local extinctions, and (re)colonizations. From these, we can derive proxies for dispersal. First, we calculated patch connectivity (*S*_*i,t*_), which is associated with dispersal potential between occupied habitat patches and thus reflects dispersal that cannot be observed from the data directly. Patch connectivity approximates the expected number of immigrants to patch *i* in year *t* as the sum of the individual immigrant contributions from all other occupied habitat patches in that particular year (Hanski, 1994). We calculated *S*_*i,t*_ similarly to Hanski et al. (2017), with the modification that the exponential dispersal kernel was corrected to account for two‐dimensional dispersal:

$$S_{i,t}=\sum_{j\neq i}A_{i}^{\mathrm{im}}\times \frac{\alpha^{2}}{2\pi }e^{-\alpha d_{i,j}}\times N_{j,t-1}$$

$A_{i}^{\mathrm{im}}$ is the area of patch *i* in hectares scaled by the exponent *im*, which represents the effects of patch size on immigration probability (Hanski, Alho, & Moilanen, 2000). $\left(\alpha^{2}/2\pi \right)e^{-\alpha d_{i,j}}$ is the two‐dimensional exponential dispersal kernel (Clark, Silman, Kern, Macklin, & HilleRisLambers, 1999), in which α is the parameter describing the scale of dispersal and *d*_*i,j*_ is the distance between patches *i* and *j* in kilometers. *N*_*j,t*−1_ is the number of observed winter nests in patch *j* in the fall of the previous year. Parameter values used in the calculation of connectivity were α  =  2 and *im* = 0.44 as estimated by Hanski et al. (2017). The value of α corresponds to a mean dispersal distance of 1 km (Nathan, Klein, Robledo‐Arnuncio, & Revilla, 2012), a value derived from *M. cinxia* mark–recapture data, population dynamics models and landscape genetic studies (Fountain et al., 2017; Hanski, Kuussaari, & Nieminen, 1994; Hanski et al., 2017). The population level connectivities were then averaged to SIN level by using weights for each patch obtained from the spatially explicit metapopulation model (see above) and log‐transformed.

Second, in addition to patch connectivity, we examined the proportion of the population within each SIN resulting from local colonization events for each study year. This describes the relative importance of colonization events for the whole SIN and estimates the part of the dispersal events that can be observed in the data (i.e., the ones that have resulted in colonization). The proportion of the population resulting from observed colonization events within each SIN was estimated as the proportion of overwintering nests found in habitat patches unoccupied in the previous spring.

Temporal trends in both connectivity and colonization were estimated with a Bayesian generalized linear mixed effects model (binomial in the former, Gaussian in the latter), with both the intercept and slope allowed to vary between SINs. For the model examining the proportion of new colonizations, we included the proportion of patches occupied in the previous year as a predictor to account for a saturation effect (i.e., if the proportion of occupied patches within a SIN is very high then there are few patches that can become newly colonized).

As the number of surveyed patches increased manifold after 1999 (see above), and as such large differences in the numbers of patches could bias analyses conducted on ratios, we decided to consider only the 2000–2015 data for our analyses of colonization and connectivity. Although the number of surveyed patches is very different pre‐ and post‐1999, the majority of the habitat patches discovered in the remapping are small and/or of low quality and therefore they contribute very little to the total number of nests, and hence to the dynamics of the metapopulation. Therefore, they are not expected to bias other analyses (Hanski & Meyke, 2005; Hanski et al., 2017).

### 2.7 Temporal trends in the parasitoid *C. melitaearum*

We examined the temporal trends in both the proportion of viable *M. cinxia* SINs that are occupied by the specialist parasitoid *C. melitaearum,* and in the proportion of *C. melitaearum* occupied patches within SINs. For the latter, to avoid zero‐inflation in the data, we used a subset of SINs that have been occupied by the parasitoid in at least eight of the study years (i.e., over a third of the study period). The former captures temporal trends in the metapopulation wide distribution of the parasitoid and the latter describes changes in the distributions within SINs. Both were analyzed using Bayesian generalized linear mixed effects models with a binomial distribution and a first‐order autocorrelation term. For the latter, intercepts and slopes were allowed to vary between SINs.

### 2.8 Implementation of statistical analyses

All statistical analyses were implemented in R (version 3.3.2; R Core Team, 2016). The linear and generalized linear mixed models were implemented using the packages brms (version 1.7.0; Bürkner, 2017) and RStan (version 2.14.1; Stan Development Team, 2016) as interfaces for the Stan statistical modeling platform (Carpenter et al., 2017). Prior to running the models, correlations between covariates and variance inflation factors were examined. In all the analyses, the variance inflation factors remained below 5, and hence, we did not consider there to be any serious multicollinearity issues. For all models, we ensured that each estimate had a minimum of 10,000 effective samples and that $\hat{R}$ values were below 1.05. In practice, this meant that for each model we ran four chains for 35,000 iterations with a warm‐up period of 5000 iterations and a thinning rate of 10 iterations.

As a weakly informative prior for the coefficients of covariates, we used a normal distribution with a mean of zero and a standard deviation of 10 in all models. For the residual standard deviation (σ_res_), we set a prior following half Student's *t* distribution with 3 degrees of freedom and a scale of 10. For the models with random effects, we set priors following a half Cauchy distribution with a scale of five for the standard deviations of the random intercepts and slopes.

Convergence and mixing of chains was inspected visually using the bayesplot R package (Gabry, 2017).

## 3 RESULTS

### 3.1 Detrending weather variables

There were temporal trends only in May temperature, March precipitation, and August precipitation, and for each of them, a single temporal basis function was selected (Figure S1, Table S3). For May temperature, the temporal basis function suggests an increasing linear trend, with temperature increasing each year by ca. 0.03 °C. For March precipitation, the smooth basis function suggests a cyclically fluctuating trend with peaks at ca. 8–9 year intervals, and for August precipitation, there is a unimodal trend, with increasing precipitation until 2008 after which precipitation has started to decline. An intercept model was selected for the rest of the weather variables.

### 3.2 Associations between population growth rate and weather conditions

*Melitaea cinxia* population growth rate is positively associated with most of the examined precipitation variables (Table 2), and negatively associated with diapause and July temperatures. No additional weather variables have coefficients differing from zero, if a credible interval (Cr.I.) of 90% is considered instead of the reported 95% interval. The strongest association is with May precipitation and the only precipitation variables not exhibiting associations with growth rate are diapause period (September to February) and August precipitations (Table 2). Our additional analyses with snow cover as a covariate suggest increasing snow cover reduces population growth rate (Table S2).

**Table 2:** Estimated coefficients, their estimated standard errors, and 95% credible intervals for the selected model on the association between weather conditions and SIN growth rates

Covariate	Est. coef.	Est. SE	95% Cr.I.	
			Lower	Upper
Intercept	−0.023	0.032	−0.086	0.040
T_D_	−0.119	0.060	−0.238	−0.001
T_Mar_	−0.058	0.057	−0.171	0.054
T_Apr_	0.026	0.048	−0.069	0.119
T_May_	0.065	0.044	−0.021	0.151
T_Jun_	0.095	0.059	−0.023	0.209
T_Jul_	−0.153	0.052	−0.254	−0.051
T_Aug_	−0.041	0.058	−0.154	0.072
P_D_	0.094	0.073	−0.049	0.239
P_Mar_	0.114	0.049	0.017	0.209
P_Apr_	0.146	0.051	0.047	0.247
P_May_	0.401	0.067	0.269	0.533
P_Jun_	0.128	0.051	0.027	0.229
P_Jul_	0.162	0.069	0.027	0.299
P_Aug_	−0.058	0.059	−0.172	0.057
AR[1]	−0.132	0.042	−0.214	−0.050
σ_(SIN intercept)_	0.051	0.039	0.002	0.143
σ_res_	0.932	0.025	0.885	0.981

AR[1]: first‐order autocorrelation term; *P*_D_: Average diapause period precipitation; *P*_Mon_: Monthly precipitation; *T*_D_: average diapause period temperature; *T*_Mon_: monthly average temperature; σ_(SIN intercept)_: standard deviation of random intercepts; σ_res_: residual standard deviation.

The above‐described coefficients derive from the full model, which best describes the relationship between population growth rate and weather conditions. However, the model for postdiapause, adult, and prediapause conditions (model number 4 in Table 1) was very similar with respect to the LOOIC value and the difference between the two models cannot be distinguished from zero (Table S4). Since the full model contains variables that have coefficients that differ from zero, but which are not in model 4, choosing the full model minimizes the risk of omitting potentially important variables from further downstream analyses.

### 3.3 Synchrony in population growth rate and weather

**Figure 1:**
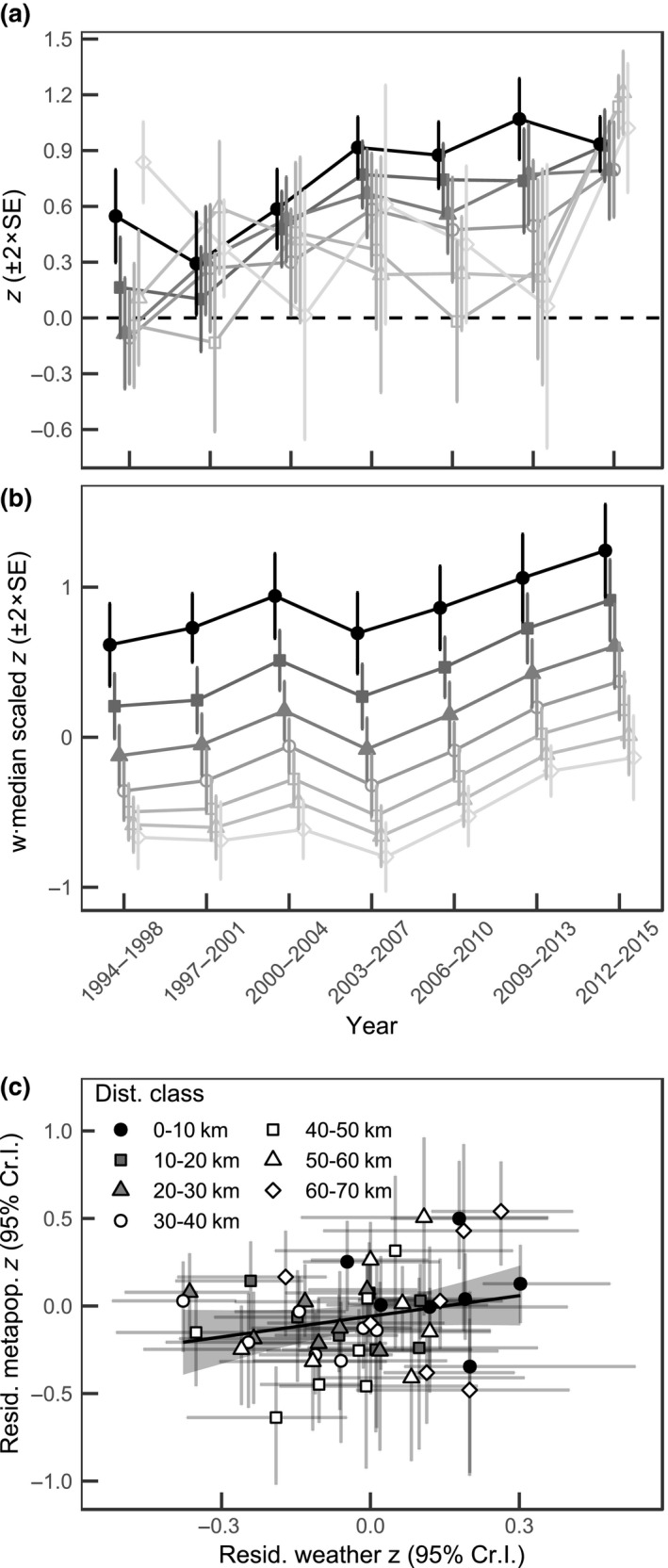
Fisher's *z*‐transformed cross‐correlation between (a) SIN annual population growth rates over different distance classes and (b) in weighted median weather conditions across time, and (c) the residual relationships between the two after accounting for distance class and temporal trend

There has been an increase in synchrony of population growth rate over time and this has occurred across different distance classes (Figure 1a). In distance classes up to 30 km, synchrony seems to be increasing until the 2003–2007 time window, after which the correlations plateau. The cross‐correlations seem to exhibit an interaction with both the time window and the distance class: the temporal trend in synchrony is stronger in shorter distance classes, whereas the temporal trend is less clear in the longer distance classes (Table 3). That being said, also the longest distance classes exhibit a clear increase in the last time window (Figure 1a). Note that the intercept refers to the first time window (1994–1998) and first distance class (0–10 km; Table 3).

**Table 3:** Estimated coefficients, their estimated standard errors, and 95% credible intervals for models of the effects of time and distance on average synchrony in SIN growth rates and weighted averaged weather conditions

Covariate	Est. coef.	Est. SE	95% Cr.I.	
			Lower	Upper
*SIN growth rate synchrony (Fisher's z‐score)*				
Intercept	0.048	0.156	−0.217	0.289
Time window	0.205	0.056	0.123	0.305
Dist. class	0.041	0.033	−0.013	0.096
Time window : Dist. class	−0.026	0.010	−0.042	−0.010
AR[1]	0.762	0.154	0.461	0.944
σ_res_	0.163	0.032	0.115	0.220
*Weather synchrony (weighted average Fisher's z‐score)*				
Intercept	0.437	0.079	0.310	0.567
Time window	0.101	0.022	0.065	0.138
Dist. class	−0.228	0.012	−0.247	−0.209
AR[1]	0.782	0.121	0.557	0.938
σ_res_	0.078	0.022	0.045	0.116
*Residual SIN growth rate synchrony vs. residual weather synchrony*				
Intercept	−0.046	0.037	−0.120	0.026
Residual weather synchrony	0.460	0.272	−0.077	0.999
σ_res_	0.189	0.035	0.125	0.262

AR[1]: first‐order autocorrelation term; σ_res_: residual standard deviation.

The synchrony of weighted average weather conditions increases with time and decreases with increasing distance class (Figure 1b, Table 3). The increase in the synchrony of weather conditions with time is similar across distances as the interaction term between time window and distance did not differ from zero (neither 95% nor 90% Cr.I; Table S5). With few exceptions, the general trend of increasing synchrony with time, especially in the two latter time windows, also holds when observing each of the weather variables separately (Figures S2 and S3).

Finally, there is a tendency for the residual population growth rate synchrony to be positively associated with residual weather synchrony (Figure 1c, Table 3). However, due to large standard errors in the estimates of the detrended residuals, the 95% Cr.I. does not differ from zero (Table 3). However, the 90% Cr.I. does not include zero (Lower: 0.022; Upper: 0.912) suggesting that a relationship between the two synchronies exists. The relationship does not seem to depend on the distance class, as the interaction between residual weather synchrony and distance class does not differ from zero (neither 95% nor 90% Cr.I; Table S6).

### 3.4 Temporal trends in population connectivity, colonization dynamics, and parasitoid distribution

**Figure 2:**
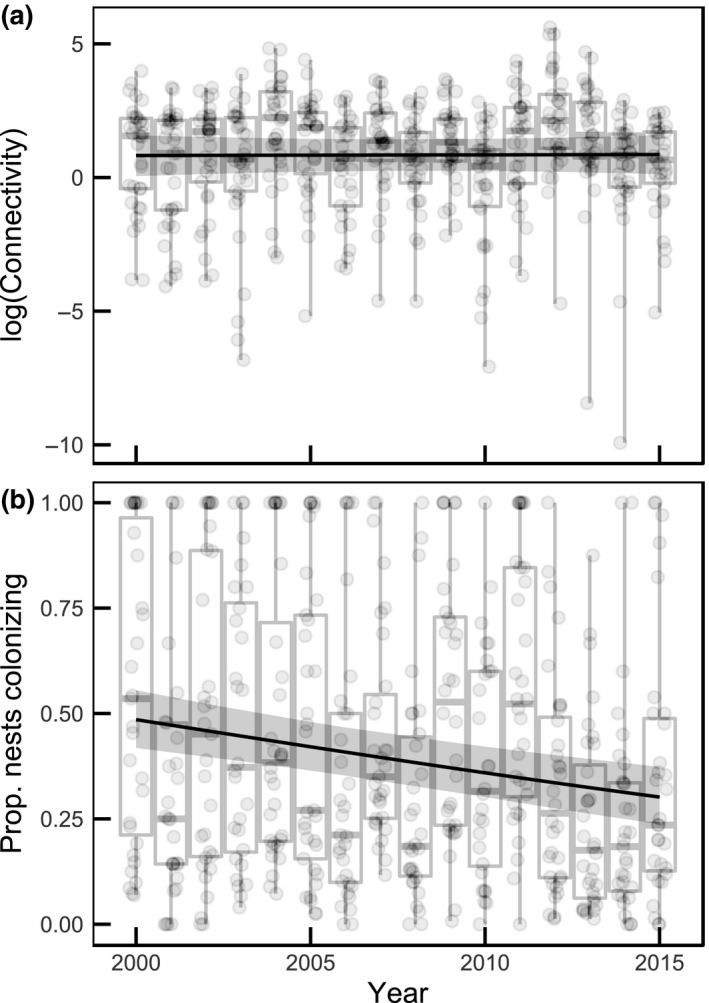
Temporal trends in (a) the connectivity between local populations and (b) the proportion of overwintering *Melitaea cinxia* nests within each SIN representing colonization of new patches. The boxplots illustrate the variability between SINs in different years and the trend line illustrates the temporal trend derived from a binomial GLM (+‐95% Cr.I.)

**Figure 3:**
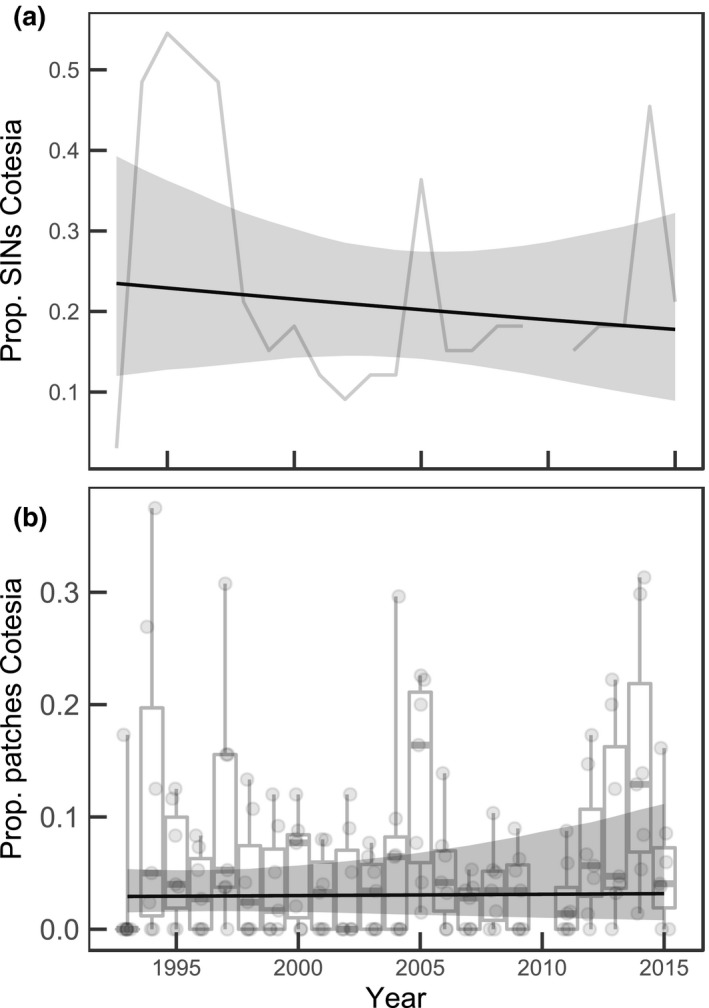
Temporal trends in the occurrence of a specialist parasitoid *Cotesia melitaearum* at the level of different (a) SINs and (b) habitat patches within SINs. The boxplots illustrate the variability between SINs in different years and the trend line illustrates the temporal trend derived from a binomial GLM (±95% Cr.I.)

We did not observe increasing trends in our proxies of dispersal over time (Figure 2, Table 4). In fact, if anything, the proportion of the population within a SIN representing colonizations of patches unoccupied in the previous time step has decreased. Similarly, there were no apparent increasing or decreasing trends in the parasitoid *C. melitaearum* distribution between or within SINs (Figure 3, Table 4).

**Table 4:** Estimated coefficients, their estimated standard errors, and 95% credible intervals for models of the temporal trends in *Melitaea cinxia* population connectivity, proportion of colonizing overwintering nests, proportion of SINs, and patches within SINs occupied by *C. melitaearum*

Covariate	Est. coef.	Est. SE	95% Cr.I.	
			Lower	Upper
*Temporal trend in log(population connectivity)*				
Intercept	0.824	0.384	0.187	1.446
Year	0.002	0.031	−0.047	0.053
AR[1]	0.412	0.064	0.310	0.522
σ_(S intercept)_	1.994	0.316	1.521	2.553
σ_(Year|SIN slope)_	0.142	0.031	0.093	0.194
σ_res_	1.227	0.044	1.157	1.303
*Temporal trend in the proportion of colonizing overwintering nests*				
Intercept	1.252	0.003	0.886	1.639
Year	−0.052	0.000	−0.080	−0.024
Prop. patches occupied_(*t*‐1)_	−5.759	0.005	−6.547	−4.971
AR[1]	0.218	0.001	0.090	0.336
σ_(SIN intercept)_	0.685	0.002	0.453	0.970
σ_(Year|SIN slope)_	0.037	0.000	0.003	0.074
σ_res_	0.975	0.001	0.890	1.070
*Temporal trend in the proportion of SINs occupied by C. melitaearum*				
Intercept	−3.505	0.005	−4.352	‐2.734
Year	0.004	0.001	−0.079	0.086
AR[1]	0.714	0.002	0.364	0.986
σ_(SIN intercept)_	0.607	0.006	0.039	1.693
σ_(Year|SIN slope)_	0.082	0.001	0.019	0.190
σ_res_	0.906	0.002	0.694	1.162
*Temporal trend in proportion of patches within each SIN occupied by C. melitaearum*				
Intercept	−1.181	0.006	−2.222	−0.287
Year	−0.016	0.000	−0.087	0.061
AR[1]	0.421	0.003	−0.125	0.927
σ_res_	0.758	0.002	0.451	1.203

AR[1]: first‐order autocorrelation term; σ_(SIN intercept)_: standard deviation of random intercepts; σ_(Year|SIN slope)_: standard deviation of random slopes of the temporal trend; σ_res_: residual standard deviation.

## 4 DISCUSSION

We observed that the previously reported temporal increase in the synchrony of the Glanville fritillary butterfly, *M. cinxia*, (Hanski & Meyke, 2005; Tack et al., 2015) metapopulation is a result of increased synchrony across all distances, and that the increase is paralleled by a temporal increase in synchrony of weather conditions (Figure 1). Furthermore, we show that other potential explanations for the increasing synchrony—namely increasing dispersal and changes in trophic interactions with an influential specialist parasitoid—do not exhibit trends matching that of increasing metapopulation synchrony, and are therefore unlikely drivers of synchrony of *M. cinxia*. We will elaborate on these below.

### 4.1 Alternative explanations for increased synchrony

An increase in synchrony could be driven by increased dispersal between local populations and SINs over time (Gyllenberg et al., 1993; Kendall, Bjørnstad, Bascompte, Keitt, & Fagan, 2000; Liebhold et al., 2004). However, ecological long‐term datasets most often do not allow for characterization of dispersal dynamics and hence disentangling the effects of dispersal and environmental conditions on population synchrony is notoriously difficult. Therefore, dispersal as a driver of population synchrony has been ruled out in situations where one can be sure that no dispersal between study regions occurs, for example, due to geographic barriers (Grenfell et al., 1998), or using comparative data on sets of species that differ in their dispersal abilities (Peltonen, Liebhold, Bjørnstad, & Williams, 2002). With extensive surveys of metapopulation dynamics (i.e., patch level extinctions and recolonizations), we can derive proxies that reflect temporal trends in dispersal within the system. We estimated both connectivity between local populations and proportion of the population representing colonizing events. Neither showed an increasing trend and, in fact, colonizations seem to have decreased over time (Figure 2 and Table 4). Hence, there is no evidence suggesting that the increased metapopulation synchrony would be associated with increased dispersal, and it may even be the opposite.

Another frequently reported driver of population synchrony is a spatially correlated predator that can drive cyclic populations into the same phase (Liebhold et al., 2004; Vasseur & Fox, 2009). Alternatively, synchrony may increase if parts of a metapopulation are released from predation as spatially restricted predator can alter the density dependence locally, creating asynchrony (Walter et al., 2017). Previous studies have documented that the braconid parasitoid wasp *C. melitaearum* can impact the population dynamics of *M. cinxia* and in some cases even drive local populations into extinction (Lei & Hanski, 1997). *Cotesia melitaearum* is rather sedentary and is typically restricted to few SINs in the *M. cinxia* metapopulation (van Nouhuys & Ehrnsten, 2004; van Nouhuys & Hanski, 2002, 2004). Therefore, any change in the distribution—be it a decrease or an increase—could potentially alter the metapopulation synchrony of *M. cinxia*. However, our results suggest no trend in either the proportion of SINs or patches within SINs related to *C. melitaearum*. Therefore, the hypothesis that synchrony could be driven by a change in density dependence due to a change in the extent of *C. melitaearum* is not supported. Admittedly, our surveys do not systematically account for natural enemies other than *C. melitaearum* so we have not analyzed them. However, the observed predators are broad generalists that do not appear to systematically use *M. cinxia* as prey (van Nouhuys & Hanski, 2004). Over many years of field studies of all life stages, we have not observed substantial changes in predator community. While they may respond to *M. cinxia* density within a local population under some conditions (van Nouhuys & Kraft, 2012), we do not expect individuals of these taxa to drive synchrony by moving in the landscape in response to local density of *M. cinxia*.

### 4.2 Evidence for a Moran effect

Population growth rate synchrony increases in parallel with the synchrony of weather conditions and, even if there is some uncertainty in the estimate, there is an indication of an association between population dynamics synchrony and weather synchrony even after the removal of the temporal trend and the effect of distance. It is worth noting that our estimate of the association is conservative as detrending synchronies with respect to time and distance prior to analyzing the relationship between them may underestimate their association (Chevalier et al., 2015). The paralleled trends in metapopulation and weather synchronies and the residual relationship between the two point toward a Moran effect, in which correlated environmental conditions force populations into synchrony (Liebhold et al., 2004; Moran, 1953).

The increase in weather synchrony can be seen in different weather components individually (Figures S2 and S3), but more importantly, it is also evident when combining the weather conditions according to their importance to *M. cinxia* population growth rate (Figure 1b). To this end, precipitation‐related variables are particularly influential for *M. cinxia* population growth rate (Table 2). Of these, May precipitation—the time corresponding to postdiapause larval development and pupation (Murphy et al., 2004)—has the strongest positive association. The importance of precipitation is concordant with a recent study suggesting a central role for precipitation in global natural selection patterns (Siepielski et al., 2017). Although in‐depth discussion of the specific mechanisms by which the different weather variables influence the *M. cinxia* metapopulation growth rate is beyond the scope of this study, the fact that May precipitation stands out as influential makes perfect sense: The larvae consume much more host plant biomass per capita during the postdiapause than during the prediapause phase, and can be forced to compete for resources with their siblings, which there can be hundreds of (Kuussaari & Singer, 2017; Kuussaari et al., 2004). As the habitats of *M. cinxia* are dry meadows characterized by shallow soils, host plant growth can be very limited in the absence of precipitation during spring.

In a previous study, Tack et al. (2015) suggested that the synchrony of *M. cinxia* in Åland was driven by an increase in the frequency of late summer drought events, while our results suggest that it is the overall increase in synchrony of weather conditions, with spring and early summer weather being most important, and late summer conditions playing less of a role (Table 2). Our analysis included many weather variables that had not been considered previously, and therefore, it is not too surprising that our findings are different from those of Tack et al. (2015). Indeed, population growth rates of several butterfly species have been reported to be sensitive to weather conditions across their life cycle (Mills et al., 2017; Radchuk, Turlure, & Schtickzelle, 2013). This being said, the results of the current study are concordant with those of Tack et al. (2015) in the sense that July precipitation was observed to be positively associated with *M. cinxia* growth rate in both.

### 4.3 Population synchrony is increasing across various systems and scales

The results at hand add to the growing pool of evidence that change in climatic conditions is likely to drive synchrony in populations dynamics across systems (Allstadt et al., 2015; Defriez & Reuman, 2017; Defriez, Sheppard, Reid, & Reuman, 2016; Hansen et al., 2013; Koenig & Liebhold, 2016; Sheppard, Bell, Harrington, & Reuman, 2015; Shestakova et al., 2016). However, few studies (if any) have reported a weather synchrony driven increase in a highly dynamic metapopulation system characterized by frequent local extinctions and recolonizations. In such dynamic systems with high local turnover, synchrony can have large effects for long‐term metapopulation viability, as habitat recolonization can be reduced due to synchronous population declines or extinctions (Hanski & Woiwod, 1993). Additionally, whether synchrony increases extinction risk or not is dependent on the source of synchrony: Increased dispersal can maintain high levels of habitat recolonization even if it increases synchrony, but environment induced synchrony is likely more detrimental as simultaneous population declines or local extinctions are less likely to be balanced out by dispersal (Hanski & Woiwod, 1993; Heino et al., 1997).

Furthermore, whereas previous studies have reported climate driven increase in population synchrony on large scale patterns ranging from regional (e.g., within the area of a country; Sheppard et al., 2015; Defriez et al., 2016; Shestakova et al., 2016) to continental (Defriez & Reuman, 2017; Hansen et al., 2013; Koenig & Liebhold, 2016), our results are at a smaller spatial scale, suggesting generalizability of the phenomenon across scales. In cyclic populations, Moran effect has been suggested to work primarily on shorter spatial scales, whereas phase locking due to dispersal and/or predators is a likely driver of synchrony at longer distances (Fox et al., 2011). Our results and the findings of Fox et al. (2011) would suggest that the impact of climate change on population dynamics can be prevalent on relatively small spatial scales.

With increasing habitat fragmentation and advancing climate change there is a need for understanding the interactions between the two, and the ways one facet of global change might influence that of the other (Holyoak & Heath, 2016; Oliver & Morecroft, 2014). Our results suggest that climate change can alter the dynamics of spatially structured populations occupying fragmented landscapes. Although the *M. cinxia* in Finland exhibits classical metapopulation dynamics with recurrent extinction and colonization events, our results should apply to other spatially structured systems with limited dispersal between local populations. Another important aspect of climate change that our results highlight is that in addition to changes in average weather conditions, changes in the spatial variability of weather conditions can have important consequences for natural populations and should not be overlooked.
