# Supplementary material for: Metapopulation dynamics in a changing climate: Increasing spatial synchrony in weather conditions drives metapopulation synchrony of a butterfly inhabiting a fragmented landscape
Source: Glob Chang Biol. 2018 May 16;24(9):4316–29. doi: 10.1111/gcb.14280 (PMC6120548; doi:10.1111/gcb.14280)
Supplement: Supplementary file 1 [file GCB-24-4316-s001.docx]

**SUPPORTING INFORMATION**

Metapopulation dynamics in a changing climate: Increasing spatial synchrony in weather conditions drives metapopulation synchrony of a butterfly inhabiting a fragmented landscape

Kahilainen, A., van Nouhuys, S., Schulz, T. & Saastamoinen, M.

**Table S1.** Annual first and last observations of adult *M. cinxia* individuals in the Åland metapopulation.

| **Year** | **First obs.** | **Last obs.** | **Flight stage duration (days)** |
| --- | --- | --- | --- |
| 1998 | June 5^th^ | July 7^th^ | 33 |
| 1999 | June 1^st^ | June 30^th^ | 30 |
| 2000 | June 5^th^ | July 6^th^ | 32 |
| 2001 | May 30^th^ | June 27^th^ | 29 |
| 2002 | May 23^rd^ | June 25^th^ | 34 |
| 2003 | June 10^th^ | July 15^th^ | 36 |
| 2004 | May 31^st^ | June 29^th^ | 30 |
| 2005 | June 15^th^ | NA | NA |
| 2006 | June 2^nd^ | July 3^rd^ | 32 |
| 2007 | May 24^th^ | NA | NA |
| 2008 | May 26^th^ | June 19^th^ | 25 |
| 2009 | June 1^st^ | July 8^th^ | 38 |
| 2010 | May 30^th^ | June 30^th^ | 32 |
| 2011 | June 7^th^ | June 17^th^ | 11 |
| 2012 | May 31^st^ | July 3^rd^ | 34 |
| 2013 | NA | June 25^th^ | NA |
| 2014 | NA | NA | NA |
| 2015 | June 3^rd^ | July 3^rd^ | 31 |

The observations are based on SvN’s personal field notes.

**Table S2.** Estimated coefficients, their estimated standard errors, and 95% credible intervals for a modification of model 1 in table 1, including average snow cover as a covariate.

| **Covariate** | **Est. coef.** | **Est. SE** | **95% Cr.I.** | |
| --- | --- | --- | --- | --- |
|  |  |  | **Lower** | **Upper** |
| Intercept | -0.022 | 0.031 | -0.073 | 0.030 |
| T_D_ | -0.156 | 0.061 | -0.256 | -0.055 |
| T_Mar_ | -0.110 | 0.061 | -0.210 | -0.009 |
| T_Apr_ | 0.032 | 0.048 | -0.047 | 0.111 |
| T_May_ | 0.056 | 0.044 | -0.016 | 0.128 |
| T_Jun_ | 0.140 | 0.061 | 0.040 | 0.238 |
| T_Jul_ | -0.145 | 0.052 | -0.230 | -0.058 |
| T_Aug_ | -0.056 | 0.058 | -0.150 | 0.039 |
| S_D_ | -0.123 | 0.041 | -0.190 | -0.056 |
| P_D_ | 0.096 | 0.073 | -0.024 | 0.216 |
| P_Mar_ | 0.121 | 0.049 | 0.041 | 0.201 |
| P_Apr_ | 0.126 | 0.051 | 0.042 | 0.209 |
| P_May_ | 0.408 | 0.067 | 0.299 | 0.519 |
| P_Jun_ | 0.121 | 0.050 | 0.039 | 0.203 |
| P_Jul_ | 0.175 | 0.068 | 0.064 | 0.285 |
| P_Aug_ | -0.074 | 0.058 | -0.168 | 0.022 |
| AR[1] | -0.142 | 0.041 | -0.210 | -0.074 |
| σ_(SIN intercept)_ | 0.040 | 0.030 | 0.003 | 0.098 |
| σ_res_ | 0.927 | 0.025 | 0.887 | 0.968 |

T_D_ = Average diapause period temperature; T_Mon_ = Monthly average temperature; S_D_ = Average diapause period snow cover; P_D_ = Average diapause period precipitation; P_Mon_ = Monthly average precipitation;AR[1] = first order autocorrelation term; σ_(SIN intercept)_ = standard deviation of random intercepts; σ_res_ = residual standard deviation

**Table S3.** Cross-validated BIC values for linear models with different numbers of orthogonal fitted smooth temporal basis functions as covariates. The models were fitted for each weather variable separately. The bolded values represent the lowest cross-validated BIC values and hence the selected model for describing the temporal trend.

| **# fun** | **T_D_** | **T_Mar_** | **T_Apr_** | **T_May_** | **T_Jun_** | **T_Jul_** | **T_Aug_** |
| --- | --- | --- | --- | --- | --- | --- | --- |
| 0 | **19.03** | **35.12** | **-2.32** | 1.48 | **13.03** | **21.39** | **16.09** |
| 1 | 20.67 | 37.36 | -1.12 | **-7.22** | 15.77 | 23.36 | 18.84 |
| 2 | 23.08 | 40.21 | 1.54 | -5.87 | 17.96 | 25.84 | 21.83 |
| 3 | 25.88 | 43.08 | 3.96 | -3.15 | 20.97 | 27.05 | 23.32 |
| 4 | 28.45 | 45.67 | 1.43 | -0.31 | 23.71 | 29.94 | 25.79 |
| 5 | 31.31 | 46.83 | 2.88 | 2.06 | 26.71 | 31.91 | 27.88 |
| **# fun** | **P_D_** | **P_Mar_** | **P_Apr_** | **P_May_** | **P_Jun_** | **P_Jul_** | **P_Aug_** |
| 0 | **111.42** | 126.58 | **121.51** | **138.48** | **130.21** | **157.72** | 157.67 |
| 1 | 112.36 | **100.36** | 124.53 | 141.18 | 131.41 | 160.37 | **155.17** |
| 2 | 114.66 | 102.55 | 126.59 | 144.13 | 133.67 | 162.88 | 157.86 |
| 3 | 116.40 | 104.71 | 129.57 | 146.73 | 135.31 | 165.73 | 159.35 |
| 4 | 117.72 | 107.16 | 132.11 | 149.68 | 134.93 | 168.49 | 162.28 |
| 5 | 119.75 | 110.20 | 132.89 | 148.98 | 137.67 | 171.31 | 164.47 |

T_Mon_ = Monthly average temperature; T_D_ = Average diapause period temperature; P_Mon_ = Monthly average precipitation; P_D_ = Average diapause period precipitation

**Table S4.** Pairwise comparisons between different models for the association between *M. cinxia* growth rate and weather conditions. The row and column labels correspond to the models in Table 1. The values above the diagonal represent the pairwise difference in the LOOIC-values of the models and the value below represents the standard error of the estimated difference. The comparison between the two of the lowest LOOIC models (models 1 and 4) is bolded.

|  | **1** | **2** | **3** | **4** | **5** | **6** | **7** | **8** | **9** | **10** | **11** | **12** | **13** | **14** | **15** | **16** | **17** |
| --- | --- | --- | --- | --- | --- | --- | --- | --- | --- | --- | --- | --- | --- | --- | --- | --- | --- |
| **1** | 0 | -25.1 | -39.7 | **0.8** | -42.4 | -129.7 | -47.9 | -25.7 | -11.4 | -50.0 | -133.4 | -53.6 | -128.2 | -58.6 | -48.8 | -22.2 | -135.6 |
| **2** | 11.9 | 0 | -14.6 | 25.9 | -17.3 | -104.6 | -22.9 | -0.6 | 13.6 | -24.9 | -108.3 | -28.5 | -103.2 | -33.5 | -23.7 | 2.8 | -110.5 |
| **3** | 13.3 | 19.9 | 0 | 40.4 | -2.7 | -90.1 | -8.3 | 14.0 | 28.2 | -10.3 | -93.7 | -13.9 | -88.6 | -19.0 | -9.1 | 17.4 | -95.9 |
| **4** | **4.0** | 11.4 | 13.7 | 0 | -43.2 | -130.5 | -48.7 | -26.5 | -12.2 | -50.8 | -134.2 | -54.4 | -129.0 | -59.4 | -49.6 | -23.0 | -136.4 |
| **5** | 15.2 | 9.3 | 21.7 | 15.3 | 0 | -87.3 | -5.5 | 16.7 | 30.9 | -7.6 | -91.0 | -11.2 | -85.8 | -16.2 | -6.4 | 20.2 | -93.2 |
| **6** | 22.9 | 19.5 | 19.3 | 22.6 | 20.5 | 0 | 81.8 | 104.0 | 118.3 | 79.7 | -3.7 | 76.1 | 1.5 | 71.1 | 80.9 | 107.5 | -5.9 |
| **7** | 14.0 | 19.8 | 7.5 | 15.0 | 21.7 | 19.7 | 0 | 22.3 | 36.5 | -2.1 | -85.5 | -5.6 | -80.3 | -10.7 | -0.8 | 25.7 | -87.6 |
| **8** | 12.3 | 4.9 | 19.2 | 12.0 | 11.6 | 18.0 | 19.6 | 0 | 14.2 | -24.3 | -107.7 | -27.9 | -102.6 | -32.9 | -23.1 | 3.5 | -109.9 |
| **9** | 9.0 | 14.7 | 15.0 | 7.9 | 14.8 | 23.4 | 15.1 | 15.2 | 0 | -38.5 | -122.0 | -42.1 | -116.8 | -47.2 | -37.3 | -10.8 | -124.1 |
| **10** | 15.5 | 20.2 | 8.9 | 14.4 | 21.5 | 20.4 | 10.4 | 20.8 | 14.7 | 0 | -83.4 | -3.6 | -78.3 | -8.6 | 1.2 | 27.8 | -85.6 |
| **11** | 23.1 | 19.6 | 20.3 | 23.3 | 18.8 | 6.2 | 19.4 | 18.9 | 22.9 | 21.3 | 0 | 79.8 | 5.2 | 74.8 | 84.6 | 111.2 | -2.2 |
| **12** | 16.7 | 12.2 | 21.6 | 16.3 | 8.3 | 19.0 | 22.1 | 11.4 | 14.4 | 22.4 | 17.8 | 0 | -74.7 | -5.0 | 4.8 | 31.3 | -82.0 |
| **13** | 22.7 | 19.2 | 19.2 | 22.1 | 19.7 | 4.0 | 19.4 | 18.5 | 22.7 | 18.9 | 6.5 | 18.9 | 0 | 69.6 | 79.5 | 106.0 | -7.3 |
| **14** | 16.2 | 20.5 | 11.2 | 15.4 | 21.7 | 20.7 | 8.4 | 20.9 | 13.9 | 6.9 | 20.5 | 22.0 | 19.3 | 0 | 9.8 | 36.4 | -77.0 |
| **15** | 15.2 | 19.5 | 13.6 | 16.4 | 20.7 | 19.0 | 14.3 | 17.9 | 15.9 | 16.7 | 19.6 | 18.8 | 19.1 | 17.0 | 0 | 26.5 | -86.8 |
| **16** | 11.8 | 12.0 | 18.6 | 11.5 | 15.3 | 21.6 | 16.4 | 12.7 | 13.3 | 17.9 | 20.9 | 16.3 | 20.8 | 16.3 | 19.6 | 0 | -113.4 |
| **17** | 23.2 | 20.0 | 20.3 | 22.8 | 18.8 | 8.0 | 19.5 | 19.4 | 21.8 | 20.0 | 5.2 | 17.3 | 6.8 | 18.8 | 19.5 | 20.6 | 0 |

**Table S5.** The full model for the association of weighted median weather synchrony with time, distance and their interaction.

| **Covariate** | **Estimate** | **S.E.** | **95% Cr.I.** | |
| --- | --- | --- | --- | --- |
|  |  |  | **Lower** | **Upper** |
| Intercept | 0.421 | 0.096 | 0.264 | 0.579 |
| Time window | 0.107 | 0.029 | 0.059 | 0.156 |
| Dist. class | -0.222 | 0.022 | -0.258 | -0.186 |
| Time window : Dist. Class | -0.002 | 0.006 | -0.012 | 0.008 |
| AR[1] | 0.783 | 0.120 | 0.558 | 0.939 |
| σ_res_ | 0.080 | 0.022 | 0.046 | 0.118 |

AR[1] = first order autocorrelation term; σ_res_ = residual standard deviation

**Table S6.** The full model for the association of residual population growth rate synchrony with residual weather synchrony, distance and their interaction.

| **Covariate** | **Estimate** | **S.E.** | **95% Cr.I.** | |
| --- | --- | --- | --- | --- |
|  |  |  | **Lower** | **Upper** |
| Intercept | 0.421 | 0.096 | 0.264 | 0.579 |
| Resid. W. synchrony | 0.107 | 0.029 | 0.059 | 0.156 |
| Dist. class | -0.222 | 0.022 | -0.258 | -0.186 |
| Resid. W. synchrony : Dist. Class | -0.002 | 0.006 | -0.012 | 0.008 |
| σ_res_ | 0.080 | 0.022 | 0.046 | 0.118 |

σ_res_ = residual standard deviation


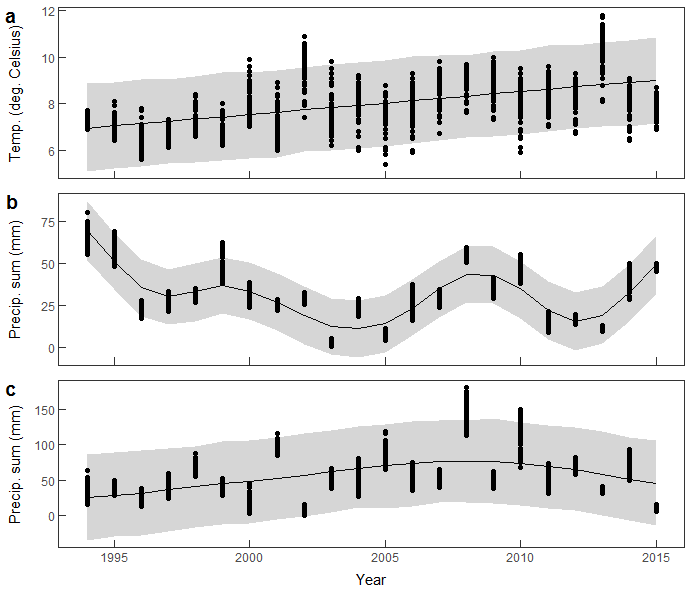


**Figure S1**. Temporal trends in (a) May temperature, (b) March precipitation and (c) August precipitation.


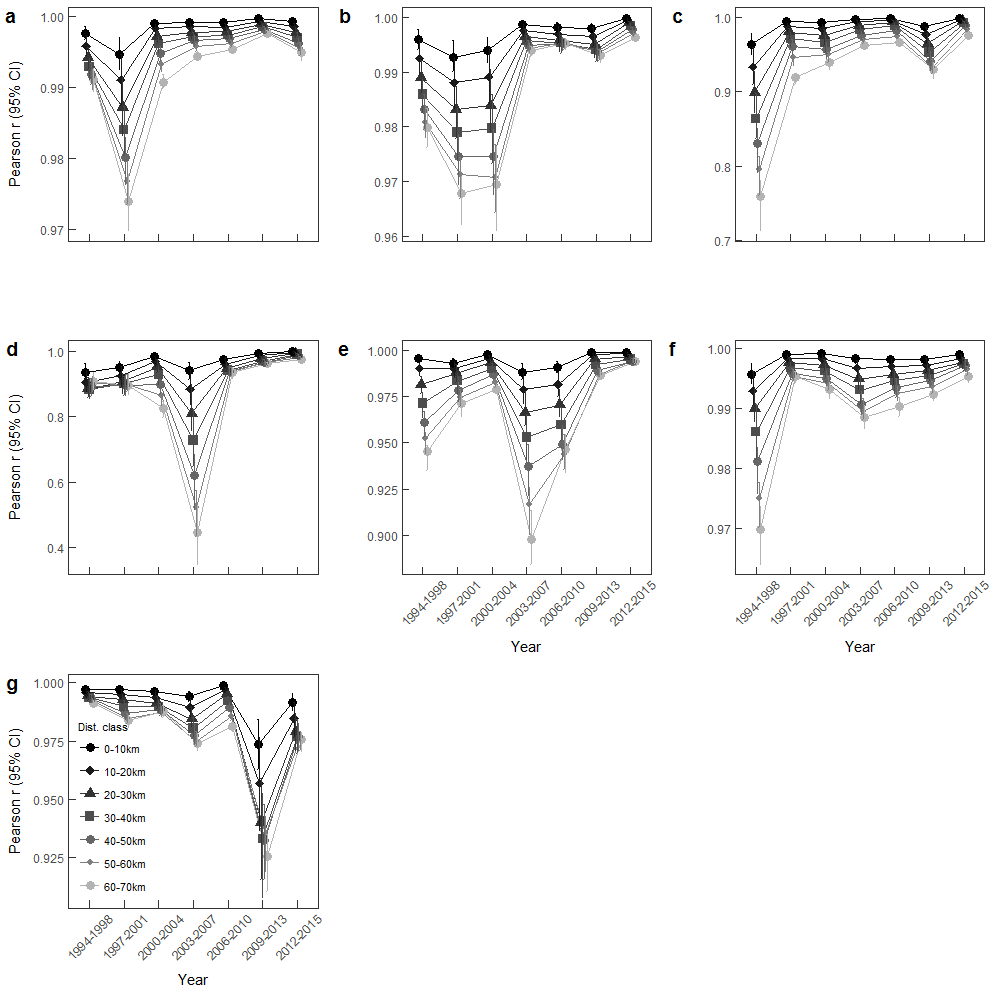


**Figure S2.** Synchrony in (a) diapause period (Sep. to Feb.), (b) March, (c) April, (d) May, (e) June, (f) July, and (g) August temperature. Note the different scales in different panels.


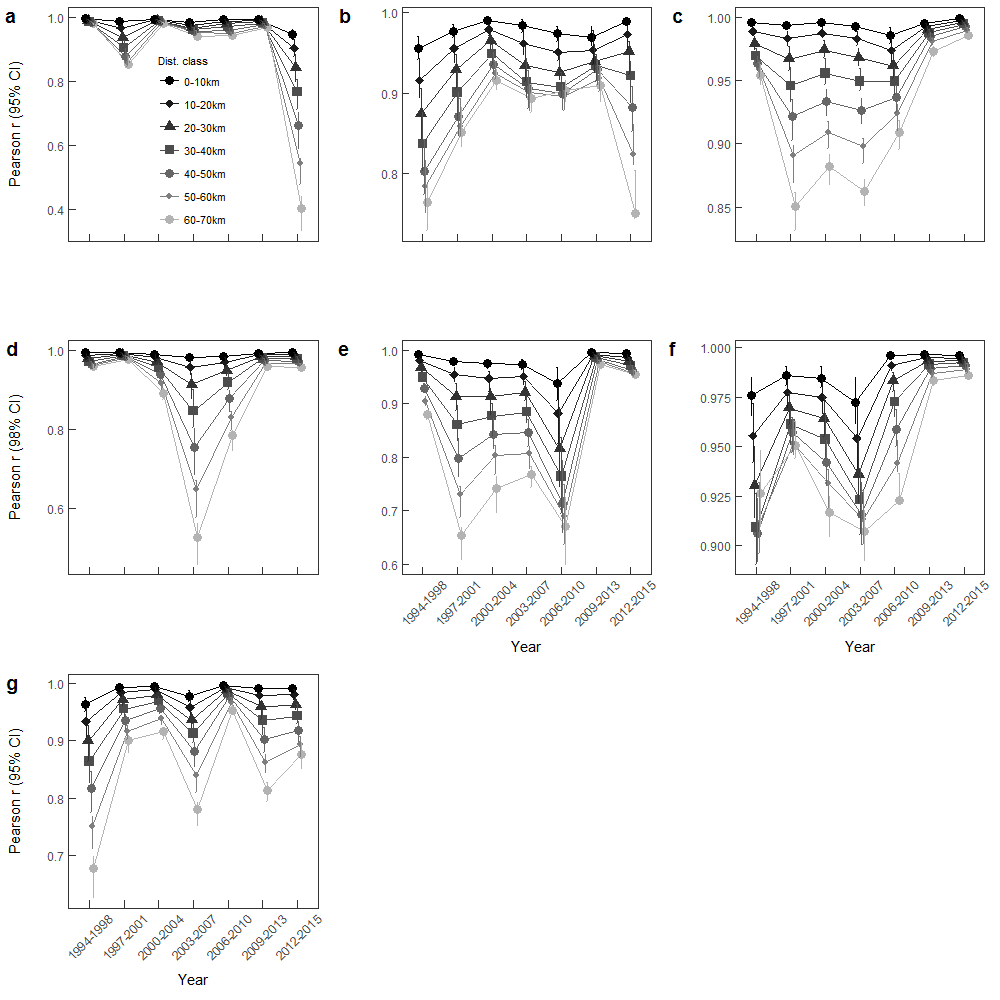


**Figure S3.** Synchrony in (a) diapause period (Sep. to Feb.), (b) March, (c) April, (d) May, (e) June, (f) July, and (g) August precipitation. Note the different scales in different panels.
